# Asthma: when suspect food allergy in patients with asthma

**DOI:** 10.1186/1939-4551-8-S1-A270

**Published:** 2015-04-08

**Authors:** Isaac Azevedo Tenorio, Aderbal Sabra, Selma Sabra

**Affiliations:** 1 Unigranrio, Brazil

## Background

The relevance of our study focuses on patient with food allergy and at increased risk for developing bronchial asthma and its association with the type of delivery, early feeding and family history of allergy.

## Methods

We analyzed randomly a study of 36 cases among the medical records of patients from the files of the BSFA (Brazilian Society of Food Allergy ), aged between 2 and 16 years, of both genders, with the diagnosis of food allergy and asthma. Asthma was diagnosed by the classical clinical picture. Food allergy was diagnosed by the normalization of the clinical picture in response to the withdraw of the offending food from the patient diet, resulting in total disapearance of the clinical disturbance. All patient relapse after the food chalenge with the offending food. All the patients had both diseases mediated by IgE.

## Results

According to age, 25 (69%) of patients were children and 11(31%) were adolecents.The first symptoms began is 4 months in 7 (19.4%) of the patients, for both diseases food allergy and asthma.

With respect to family history of food allergy was observed only in 1st degree relatives, 26 among the mothers (72%), 24 among the fathers (66%) and 7 in brothers (19,4%).

Regarding the gestational history there has been a significant number of patients delivered by cesarean section 31 (86.1%). Only 3 (8.35%) were normal delivery.

The results of analysis concerning early feeding history shown that regard to breastfeeding only 18 (50%) patients were breastfed, 15 (41.66%) did not know and 3 (8.33%) were not.. From the total number of records analyzed, 24 (66.65%) introduced some type of formula before six months of life

## Conclusions

The idea that we want to bring is article is the association among food allergy and asthma and the 3 major clinical relevant data used to the diagnosis of FA: family history of allergy, early introduction os formula and absence of normal delivery (excess of hygiene at cesarian delivery).

